# Mapping O- and N-Glycosylation in Transmembrane and Interface Regions of Proteins: Insights from a Database Search Study

**DOI:** 10.3390/ijms26010327

**Published:** 2025-01-02

**Authors:** Giorgiana Diana Carmen Anghelescu, Maria Mernea, Dan Florin Mihăilescu

**Affiliations:** 1 Doctoral School in Biology, Faculty of Biology, University of Bucharest, 91–95 Splaiul Independenței Str., 050095 Bucharest, Romania; g.anghelescu20@s.bio.unibuc.ro; 2 Department of Anatomy, Animal Physiology and Biophysics, Faculty of Biology, University of Bucharest, 91–95 Splaiul Independenței Str., 050095 Bucharest, Romania; dan.mihailescu@bio.unibuc.ro

**Keywords:** transmembrane proteins, O-glycosylation, N-glycosylation, database search, glycan mapping

## Abstract

Glycosylation is a critical post-translational modification that influences protein folding, stability and function. While extensively studied in extracellular and intracellular regions, glycosylation within transmembrane (TM) regions and at membrane interfaces remains poorly understood. This study aimed to map O- and N-glycosylation sites in these regions using a comprehensive database search and structural validation where possible. Extensive database searches revealed glycosylation sites in a range of membrane proteins. Only the sites falling in the TM regions and at the membrane interface (according to Uniprot annotations) were retained. The location of these sites was confirmed based on available 3D structures. We identified 32 O-glycosylation sites and 7 N-glycosylation sites in the TM domains of 29 proteins. O-GlcNAc sites validated as located within TM regions presented side chains either oriented toward the lipid bilayer or buried within the protein. N-glycosylation sites predicted in protein TM regions were largely confined to interface or extracellular domains. The results obtained here highlight the occurrence of glycosylation in TM regions of proteins and at membrane interfaces. This dataset provides a valuable foundation for the further exploration of structural and functional roles of glycosylation in membrane-associated regions.

## 1. Introduction

Glycosylation, namely the covalent attachment of carbohydrates, is one of the most abundant and diverse post-translational modification of proteins [1]. Protein glycosylation plays significant roles under physiological conditions, including embryogenesis, cell adhesion, signal transduction, protein folding and modulation of the immune system [2,3,4]. Some of the pathologies associated to aberrant glycosylation are chronic inflammation, autoimmunity [4], cancer development and metastasis [2].

There are different categories of protein-attached glycans, but the most common types are O-linked to Ser of Thr residues [5,6] or N-linked to Asn residues [7]. N-glycosylation occurs in nascent proteins, assisting the folding of the protein and the maturation in endoplasmic reticulum (ER) and Golgi compartments [7] and also ensuring quality control [8].

O-glycosylation can involve the attachment of different types of sugars like O-linked *N*-acetylgalactosamine (O-GalNAc), O-linked *N*-acetylglucosamine (O-GlcNAc), O-linked fucose, mannose or glucose. The most abundant types of O-glycans are O-GalNAc and O-GlcNAc [9]. O-GalNAcylation may occur in ER and Golgi compartments after the folding of the protein is complete, which allows only the glycosylation of Ser and Thr residues exposed at the surface of the protein [5]. O-GlcNAcylation resides in the cytoplasm, nucleus and mitochondria, affecting almost all functional classes of proteins [6]. O-GlcNAcylation is a noncanonical, ubiquitous and highly dynamic glycosylation, functioning as a nutrient-sensing mechanism in cells [9]. In addition, it influences the activity of many proteins, and the O-GlcNAc pattern appears to be connected to the activation of T cells [10] or to the Warburg effect in cancer cells [11].

Membrane proteins are extremely important components of the plasma membrane, being involved in mediating transportation across the plasma membrane, enzymatic reactions, signal transduction, and cell-to-cell communication and interactions [12]. Because glycosylation modulates the structure, interaction and functions of proteins, the characterization of glycans linked to membrane proteins is of great interest [13]. While the glycosylation of extracellular regions of proteins is extensively studied [14,15,16], the presence and functional implications of glycosylation within transmembrane (TM) regions and at membrane interfaces remain underexplored. By inquiring publicly available databases on proteins and experimentally validated datasets on protein glycosylation, here, we identified O-glycosylation and N-glycosylation sites in TM regions and their adjacent interfaces. By mapping these sites across a diverse protein dataset, we aim to provide foundational insights into their prevalence and distribution. In this way, we draw attention to the consideration that glycosylation sites might exist in the membrane, and we offer a starting point for future experimental and functional investigations addressing this phenomenon.

## 2. Results

### 2.1. Glycosylation Sites in TM Regions

We identified 29 proteins with reported O- or N-glycosylation in TM regions (Table 1 and Table S1). There are 24 proteins for which 32 O-GlcNAcylation sites were reported in TM regions: (i) 17 proteins presented a single site, (ii) 6 proteins presented two sites and (iii) 1 protein presented three sites. In the case of N-glycosylation, we identified seven sites in the TM regions of seven proteins, one per protein. As shown in Table 1, there are unique proteins bearing the two types of glycosylation, except for protein patched homolog 1 and the non-selective voltage-gated ion channel VDAC1 that present both O- and N-glycosylation in the TM regions. Additionally, we identified two cases of predicted glycosylation sites (2 O-glycosylation sites) that should fall in TM regions. The O-linked glycans present at TM sites were determined experimentally and are represented by O-GlcNAc (Table S1). The N-linked glycans at identified TM sites are mostly not reported, with the few exceptions being shown in Table S1.

To confirm the positioning of the glycosylation sites in TM regions and to show how the glycans can be incorporated in the structures, we searched for the 3D structures of identified proteins. We retrieved 3D structures from the Protein Data Bank for 12 proteins and used these to assess the topology of regions comprising the glycosylation sites (Table S2). Results will be detailed in the following sub-sections. For eight proteins without experimentally determined structures, we identified AlphaFold models in which the TM regions were predicted with confidence (pLDDT > 70). For nine proteins, no accurate structural data were available. From these, butyrophilin subfamily 3 member A1, HLA class II histocompatibility antigen gamma chain, antigen peptide transporter 1, ryanodine receptor 3, polypeptide N-acetylgalactosaminyltransferase 14 and E3 ubiquitin-protein ligase MARCHF5 present a single O-GlcNAcylation site; protein GOLM2 presents a single N-glycosylation site; E3 ubiquitin-protein ligase MARCHF1 presents 2 O-GlcNAcylation sites on the same TM helix; and ATP-binding cassette sub-family A member 13 presents 3 O-GlcNAcylation sites on the same TM helix.

To improve the assessment on the location of identified TM glycosylation sites in the proteins with unknown crystal structures, we used several tools to predict the topology of their surrounding residues based on the amino acids sequence (Table S2). We observed that, in most cases, there is a good agreement between the predictions in locating the glycosylation sites in TM regions. There are a few cases in which the predictions disagree. For instance, residue Ser194 from solute carrier family 25 member 53 or residues Thr132 and Thr133 from the olfactory receptor 6K3 are predicted by Uniprot and two platforms to be on TM helices, while five other platforms predict them not to be found on TM helices. Also, we should mention that six out of the seven platforms that we used in addition to Uniprot fail to identify TM beta strands, like in the case of the non-selective voltage-gated ion channel VDAC1 and the voltage-dependent anion-selective channel protein 3.

#### 2.1.1. Orexin/Hypocretin Receptor Type 1

**Figure 1:**
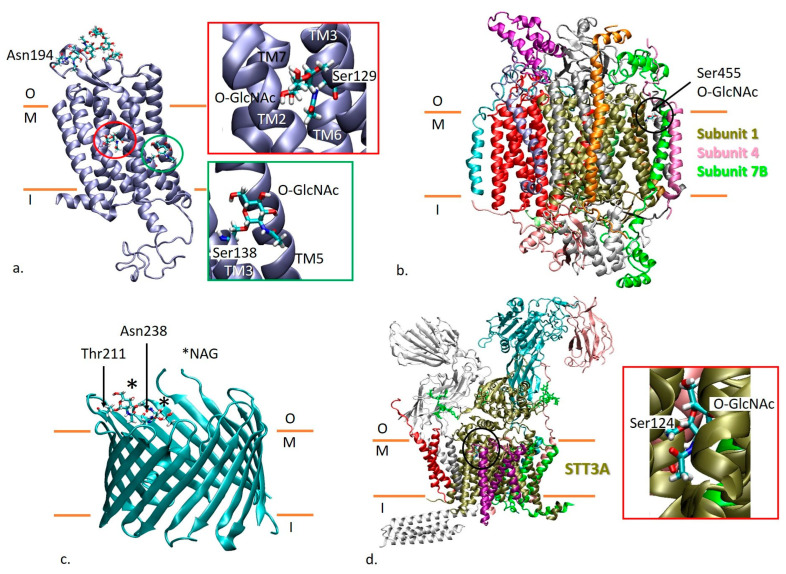
(**a**) The model of orexin/hypocretin receptor type 1 (6TO7 structure [17]) in ice blue with modeled O-GlcNAcylation at residues Ser129 and Ser138. (**b**) Cytochrome c oxidase structure (PDB id 5Z62 [18]) comprising the subunit 1 (in tan) with a modeled O-linked glycan at Ser455. The subunits of cytochrome c oxidase are shown in different colors. (**c**) The structure of non-selective voltage-gated ion channel VDAC1 (PDB id 5XDN [26]) in cyan with glycans modeled at Thr211 and Asn238. (**d**) The structure of human oligosaccharyltransferase complex OST-A (PDB id 6S7O [27]) colored according to its subunits, comprising the subunit STT3A (in tan) with a modeled O-linked glycan at Ser124. The membrane is represented as two orange lines that mark the regions outside of the cell or cell compartment (O), in the membrane region (M) and inside of the cell or cell compartment (I). Details on the location of modeled glycans are given in inserts.

In the case of orexin/hypocretin receptor type 1, Woo et al. [10] identified the presence of two O-GlcNAcylation sites at residues Ser129 and Ser138. Residue 129 is found on the TM3 helix, in the middle of the membrane, with the side chain being oriented toward the interface of TM helices and being close to the binding site of ligands such as suvorexant [17]. The glycan modeled at this site based on 6TO7 structure [17] extends in the space lined by helices TM2, TM6 and TM7 (Figure 1a). The side chain of residue 138 of TM3 helix is facing the lipid bilayer, so the modeled glycan faces the lipid bilayer. At the same time, it is close to residues from TM5 helix (Figure 1a).

#### 2.1.2. Cytochrome c Oxidase Subunit 1

Cytochrome c oxidase subunit 1 is a component of cytochrome c oxidase, the 14-subunit enzyme in the mitochondrial electron transport chain [18]. According to the 3D structure 5Z62 [18], the subunit 1 is at the core of the complex. Residue Ser455, reported as an O-GlcNAcylation site [10,19], is found on the upper side of the XII TM helix at the interface with subunit 4 (pink) and subunit 7B (green) (Figure 1b). There are no lipids at this particular interface. The glycan modeled at Ser455 extends at the interface with the same subunits.

#### 2.1.3. Non-Selective Voltage-Gated Ion Channel VDAC1

For the non-selective voltage-gated ion channel VDAC1, there are two glycosylation sites found at residues Thr211 (O-linked) [11,20,21,22,23,24] and Asn238 (N-linked) [25]. Both residues appear to be the last residues from two different TM beta strands (Uniprot topological domains annotation). According to OPM and PDBTM interrogated for the 5XDN structure, residue Thr211 is found outside of the membrane, while Asn238 is in the membrane at the end of a TM beta strand (Table S2). In Figure 1c, we show the structure of VDAC1—PDB code 5XDN [26] with a NAG residue attached at each site. The side chains of both Thr211 and Asn238 residues are oriented toward the pore. Thus, modeled glycans are also oriented toward the pore.

#### 2.1.4. Dolichyl-Diphosphooligosaccharide-Protein Glycosyltransferase Subunit STT3A

STT3A is part of the human oligosaccharyltransferase complex OST-A [27]. Several studies [10,22,23,28,29,30,31,32] reported an O-GlcNAcylation at Ser124. According to the 6S7O structure [27], the residue is found on the upper side of a TM helix (residues 118–139) and is located in the hydrophobic region of the membrane, with the side chain being oriented toward the neighboring TM helices and in the opposite direction of the lipids found at the interface between STT3A, dolichyl-diphosphooligosaccharide-protein glycosyltransferase subunit 1 and transmembrane protein 258. The modeled glycan is confined between the TM helices of STT3A spanning between residues 118–139, 10–35, 140–152, 154–160 and 168–190 (Figure 1d).

#### 2.1.5. C-C Chemokine Receptor Type 3 (CCR3)

**Figure 2:**
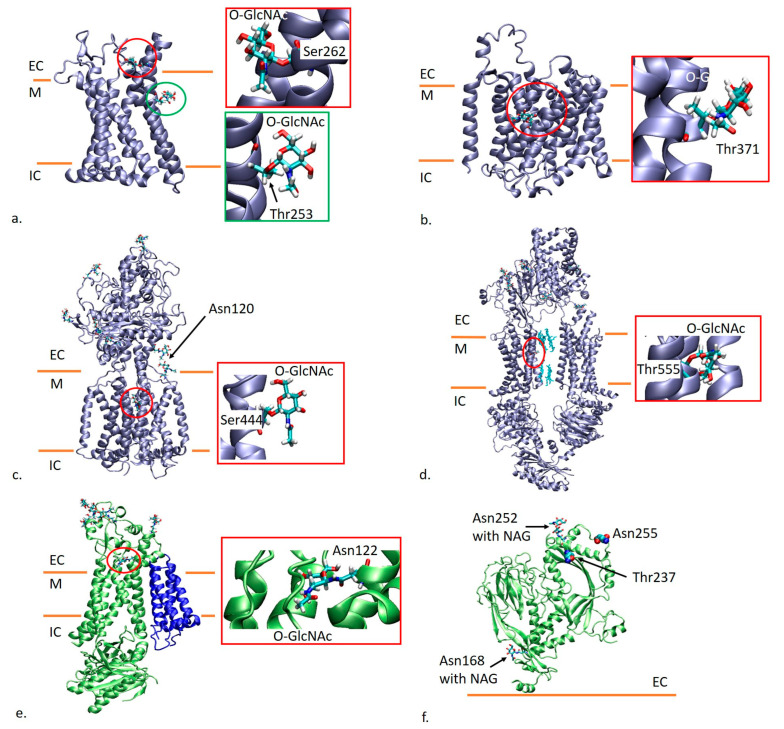
(**a**) The structure of C-C chemokine receptor type 3 (PDB id 7X9Y [33]) colored in ice blue with O-glycans being modeled at Ser262 and Thr253. (**b**) Ammonium transporter Rh type A (PDB code 8CSX [34]) colored in ice blue with an O-glycan modeled at Thr371. (**c**) Protein patched homolog 1 (PDB id 6DMB [35]) colored in ice blue with a modeled O-linked glycan at Ser444. Additionally, Asn120 was highlighted. (**d**) Structure of phospholipid-transporting ATPase ABCA7 (PDB id 8EDW [36]) colored in ice blue with an O-linked glycan modeled at Thr555. The phosphothiophosphoric acid-adenylate ester residues found in the ligand binding pockets are shown as cyan licorice. (**e**) Structure of cytochrome b-245 comprising light (blue) and heavy (green) chains (PDB id 8GZ3 [37]). The heavy chain was glycosylated at Asn122. (**f**) The structure of one chain of macrophage-expressed gene 1 protein from the pre-pore forming complex, PDB id 6U2J [38] (colored in green). The glycosylation present in the structure is labeled. Additional predicted glycosylation sites (Asn255 and Thr237) are highlighted. The membrane is represented as two orange lines that mark the regions outside of the cell or cell compartment (O), in the membrane region (M) and inside of the cell or cell compartment (I). Details on the location of modeled glycans are given in inserts.

Two O-GlcNAcylation sites identified in the case of CCR3 at residues Ser262 and Thr253 [10] are found in the TM region of the receptor on the sixth TM helix. Figure 2a shows the receptor with modeled glycans. In what concerns Ser262, the residue is close to the extracellular end of the TM helix [33]. According to the records of OPM and PDBTM on this protein (PDB code 7X9Y [33]), the residue is found at the interface with the membrane, being located outside of the membrane (Table S2). The modeled glycan extends toward the ligand binding pocket of the receptor. On the other hand, Thr253 is found in the membrane, and its side chains are oriented toward the lipid bilayer. The modeled glycan extends toward the lipid bilayer, also being close to residues from the fifth TM helix of CCR3.

#### 2.1.6. Ammonium Transporter Rh Type A (RhAG)

RhAG was reported to present an O-GlcNAcylation site at residue Thr371 [10]. Two RhAG subunits and a blood group Rh (CE) polypeptide (RhCE) form trimers present in the erythrocyte membrane [34]. According to the available 3D structures [34], the residue Thr371 of RhAG is found in the middle of the TM helix spanning between residues 357 and 384. It faces the lipid bilayer, being far from the subunit interfaces in the trimer. Thus, in Figure 2b, we present a single RhAG monomer (PDB code 8CSX [34]) with modeled glycosylation. The glycan is found at the interface with the lipid bilayer, being at the same time close to a neighboring helix (residues 137–162).

#### 2.1.7. Protein Patched Homolog 1

There are two reports [39,40] showing that protein patched homolog 1 presents an O-GlcNAcylation at residue Ser444. This residue is found on a TM helix of the protein (residues 439–457), being located in the upper half of the helix according to the 6DMB structure [35]. The side chain of Ser444 faces the lipid bilayer and thus the modeled glycan faces the lipid bilayer as well without interacting with other TM helices from protein patched homolog 1 (Figure 2c). We should note that the protein is also N-glycosylated in the extracellular regions, with six NAG residues being attached to Asn 141, 312, 349, 414, 875 and 1000 [35]. Additionally, the GlyGen database annotated a N-glycosylation site for protein patched homolog 1 at Asn120 (Figure 2c). The evidence was represented by the 3D structures 6DMB, 6DMO and 6DMY [35]. Upon careful inspection, there is no glycan reported at this site in the PDB structures. Also, we mention that Asn120 is considered extracellular by OPM and PDBTM, while it is annotated as transmembrane in the Uniprot topological domains (Table S2).

#### 2.1.8. Phospholipid-Transporting ATPase ABCA7

ABCA7 appears to present an O-GlcNAcylation site at residue Thr555 [10,41] located on the TM helix between residues 541 and 575. ABCA7 is a large protein of 2146 amino acids. The particular TM helix bearing the O-glycosylation is part of the first TM domain (TMD1) according to the structures solved by Le et al. [36]. The side chain of Thr555 extends toward the interface with the neighboring helix (residues 652–666) and is found within 10 Å of an AGS residue (phosphothiophosphoric acid-adenylate ester) from the TMD luminal space involved in lipid transport. The glycan modeled at this site faces the neighboring helix and the TMD lumen, being even closer to AGS residues (Figure 2d).

#### 2.1.9. Chloride Intracellular Channel Protein 1 (CLIC1)

CLIC1 was reported to present a N-linked glycan at Asn42 [42,43]. According to the annotation of TM helices in Uniprot based on the sequence analysis, Asn42 is part of the helix ranging from residue 26 to 46, with the note “after insertion into the membrane”. CLIC1 exists in both soluble and integral membrane forms [44]. The 17 structures of CLIC1 available in Protein Data Bank represent soluble forms of CLIC1. A single structure, namely 1RK4 [45], is annotated as a membrane protein, and OPM labels this structure as monotopic/peripheral. Thus, the structure of CLIC1 inserted into a membrane is unknown.

#### 2.1.10. Cytochrome b-245 Heavy Chain

The 3D structures of cytochrome b-245 heavy chain in complex with cytochrome b-245 light chain solved by Liu et al. [37] showed that the heavy chain presents N-linked glycans at residues 132, 149 and 240, all situated in the extracellular region of the chain. In addition, Deeb et al. [43] identified an additional N-glycosylation site at residue Asn122. According to the Uniprot annotation of transmembrane regions based on sequence analysis, the residue is found in a TM helix between residues 103 and 123. The 3D structure 8GZ3 [37] shows that Asn122 is indeed located on a helix comprising residues 104 and 128. OPM and PDBTM indicate that Asn122 is found in a region of the helix that partitions outside of the membrane (Table S2). The side chain of Asn122 is oriented toward the interface with the extracellular regions of neighboring TM helices: residues 173–192 and residues 50–69. The modeled glycan is confined between these helices (Figure 2e).

#### 2.1.11. Sodium Channel Protein Type 4 Subunit Alpha

A N-glycosylation site of sodium channel protein type 4 subunit alpha at Asn1162 was annotated by GlyGen as a reported site. The source of this annotation was the Protein Data Bank structure 6AGF [46]. Upon careful inspection of the structure, we did not find a glycosylation site at residue Asn1162.

#### 2.1.12. Macrophage-Expressed Gene 1 Protein (MPEG-1)

Two studies [25,43] report that MPEG-1 presents a N-glycosylation site at Asn255. According to Uniprot, Asn255 lies on the helix ranging between residues 248 and 256 that fold as a beta strand. MPEG-1 forms hexadecameric assemblies that initially form prepore complexes. Upon acidification, these complexes form pores in the membranes of engulfed bacterial targets [38]. Regarding the positioning of MPEG-1 relative to the lipid bilayer, OPM labels the protein as monotopic/peripheral and shows the prepore complex (6U2J structure) as attached to the outer side of the membrane. Asn255 is found on the opposite side of the protein relative to the lipid bilayer. It is worth mentioning that in the 6U2J structure [38], MPEG-1 is glycosylated at Asn168 and Asn252, the latter being very close to Asn255 (Figure 2f). In the case of MPEG-1, there is a predicted O-glycosylation site at Thr237, predicted to be located on the same TM helix as Asn252. As Figure 2f shows, this site is also located opposite to the membrane.

### 2.2. O-Glycosylation Sites at the Intetrface with the Membrane

We identified four proteins with O-glycosylation sites at the interface with the membrane (Table 2 and Table S3). The only protein that has a known 3D structure is the copper-transporting ATPase 2 (Uniprot ID P35670). Our database search identified this protein as having an O-glycosylation site at the interface with the membrane based on the Uniprot annotation of topological domains, suggesting that residues 765–785 form a TM helix (annotation performed by manual assertion according to sequence analysis). As revealed by the electron microscopy study of Yang et al. [47], residue T788 is found next to a helix spanning between residues 766 and 787. According to OPM and PDBTM, the helix partially locates into the membrane (residues 766–781) and partially into the cytoplasm (residues 782–787). Thus, residue T788 is found in the cytoplasm, close to the membrane, but not at the interface with the membrane. It is interesting to note that a variant involving this glycosylation site, namely T788I, is pathogenic, being related to Wilson disease (see Table S3 for more details).

There are no 3D structures available in the case of the three other proteins that presumably present O-glycosylation sites at the interface with the membrane (lysosome-associated membrane glycoprotein 1, neuropeptides B/W receptor type 1 and plasma membrane calcium-transporting ATPase 4). The additional topological domains predictions that we performed based on the amino acid sequences of these proteins (Table S3) support the extramembrane location of these sites and the positioning at the membrane interface.

### 2.3. N-Glycosylation Sites at the Intetrface with the Membrane

We identified 10 proteins that are expected to present N-glycosylation sites at the interface with the membrane (Table 3 and Table S4). In this search, nine glycosylation sites were predicted by Uniprot based on sequence analysis, and one site was identified from a publication. The location of these sites relative to the membrane was also assessed based on the topological domains annotated under the subcellular localization section in Uniprot and was verified based on the available 3D structures.

From these structures, we confirm the location of predicted binding sites at the interface or close to the membrane in the case of seven proteins. No glycans attached at these sites are found in the 3D structures of proteins.

For one protein, namely the sodium channel protein type 10 subunit alpha, we observed that the annotation of topological domains in Uniprot does not agree with the topology of the 3D structure according to OPM and PDBTM (Table S4). In this case, residue Asn1312 (the predicted N-glycosylation site) is located in the extracellular region, far from the interface with the membrane. Even more, in the electron microscopy structures 7WE4, 7WEL, 7WFR and 7WFW [48], there is an NAG residue linked to Asn1312.

In the case of leukocyte surface antigen CD47 and protein unc-93 homolog B1, the inspection of available 3D structures revealed that the predicted glycosylation sites are located on TM helices (see Table S4). The X-ray diffraction structure of leukocyte surface antigen CD47 (PDB id 7MYZ [49]) comprises the sequence of the mature protein (PRO_0000014880) resulting after the removal of the 18 residues signal peptide from the product of CD47 gene, and the mature protein sequence was renumbered by one. Thus, residue Asn206 (predicted glycosylation site by Uniprot) corresponds to Asn188 from the 3D structure. Asn188 is located on a TM helix (residues 186–207 identified from the protein membrane localization given by PDBTM) close to the membrane interface.

Uniprot annotation of topological domains (by sequence analysis) of protein unc-93 homolog B1 shows that residues 428–448 represent a TM helix, making Asn449 a predicted glycosylation site at the interface with the membrane. The membrane localization of protein unc-93 homolog B1 given by PDBTM according to 7CYN, 7C76 and 7C77 structures [50] shows that residues 434–455 represent a TM helix (Table S4). Thus, residue Asn449 is located on a TM helix and is not close to the membrane interface.

The importance of residues representing possible glycosylation sites is high in the case of ammonium transporter Rh type A, where the mutation that substitutes Asn355 by Ser was identified as a somatic mutation in liver cancer (according to COSMIC database). Also, the substitution of Asn206 by Ser in leukocyte surface antigen CD47 was identified as a somatic mutation in bladder urothelial carcinoma (COSMIC database).

## 3. Discussion

In the present paper, we investigated the distribution of O- and N-glycosylation sites in membrane sparing regions or in membrane interface regions of proteins. The glycosylation of proteins is important for regulating protein functions (folding, stability, phase separation), for regulating cell adhesion, for mediating the immune evasion in the case of parasites and cancer cells and for modulating signal transduction or regulating the metabolism [3]. Abnormal glycosylation was seen in the case of human diseases, including hematologic malignancies (leukemia, lymphoma, plasma cell diseases, myeloproliferative neoplasms), solid malignancies (respiratory neoplasms, gastrointestinal tumors, reproductive cancers, breast cancer, central nervous system tumors, sarcoma), autoimmune diseases, cardiovascular diseases, metabolic diseases or neurodegenerative diseases [3].

### 3.1. The Impact of Database Annotations on the Identification of Glycosylation Sites

The search for TM glycosylation sites was based on the datasets from the GlyGen database [51]. The database comprises information that was compiled, harmonized and integrated from diverse resources, including National Center for Biotechnology Information (NCBI), UniProt, the Protein Data Bank (PDB), UniCarbKB, and the GlyTouCan glycan structure repository [51]. Even more, GlyGen, along with GlyCosmos and Glyco@Expasy, are part of the GlySpace Alliance, an initiative aiming to provide the same basic dataset of glycan-related omics data through the unique portals with specific user interfaces [52]. Taking these into consideration, plus the ease of accessing the datasets, we conducted our search for glycosylation sites mainly in GlyGen. Using only GlyGen, we identified a large number of glycosylation sites in the TM regions of proteins. There remains a small possibility that some glycosylation sites, which may be underrepresented in GlyGen, were not captured in this study.

To identify the glycosylation sites in the membrane and at the membrane interface, the results from GlyGen were filtered based on the topological data annotated in Uniprot (subcellular localization section). With some exceptions, the Uniprot annotations on topological domains found in the proteins from Tables S1–S4 were performed by manual assertion according to sequence analysis. Errors in predicting the topological domains could lead to errors in the classification of identified glycosylation sites. For checking the reliability of predictions in Uniprot, we used additional platforms to predict the topology of protein regions with glycosylation sites, and we checked the location of residues in available 3D structures. With a few exceptions presented in Tables S2 and S3, there is a good agreement between the topology prediction in Uniprot and in the other seven prediction platforms that we used. Nevertheless, the best validation of topology is performed using experimentally determined 3D structures, but such information is not available in the case of all proteins identified here.

The location of identified glycosylation sites was checked in the case of proteins with known 3D structure and membrane localization predicted by OPM and PDBTM databases. We found a good agreement between the expected location and the actual location of identified glycosylation sites. There are a few exceptions in the cases of: (i) VDAC1—Thr211, expected to be found in the TM, is located on a beta strand that crosses and exits the membrane, the residue being found outside the membrane; (ii) CCR3—Ser262, expected to be found in the TM, is found on a helix that crosses the membrane, and the residue is located on the interface with the membrane, being marked as extracellular by PDBTM; (iii) protein patched homolog 1—Asn120, expected to be found in the TM, is located outside of the membrane; (iv) cytochrome b-245 heavy chain—Asn122, expected to be found in the TM, is located on the extracellular region of a helix that crosses the membrane and exits; (v) MPEG-1—Asn255, expected to be found in the TM, is found outside of the membrane, with the protein being marked as peripheral; (vi) sodium channel protein type 10 subunit alpha—Asn1312 is not found at the interface with the membrane; (vii) leukocyte surface antigen CD47—Asn206 is found in the TM region of the protein; and (viii) protein unc-93 homolog B1—Asn449, expected to be found at the interface with the membrane, is located in the TM region of the protein. The discrepancies in the TM domain boundaries definitions between OPM and PDBTM may occasionally influence site inclusion, which can be seen in the case of predicted N-glycosylation sites at the interface with the membrane (Table S4). Still, no such cases were seen in the assessment of TM glycosylation sites.

Despite the potential sources of bias in our study, we successfully identified glycosylation sites that are undoubtedly located within the membrane, as confirmed by the location in the 3D structures. In addition, we identified a larger number of glycosylation sites potentially located in the membrane that still need experimental validation.

### 3.2. The Proteins with Identified TM Glycosylation Sites

The list of proteins with O- and N-glycosylation sites located in the membrane are given in Table 1. The length of these proteins varies from 241 amino acids (CLIC1) to 5058 amino acids (ABCA13). The majority of identified proteins present 251–500 residues (15 proteins) or 501–750 residues (7 structures), as shown in the histogram in Supplementary Material S5, Section S1. The records of Uniprot comprise the oligomerization of 14 from the 29 proteins in the list (Supplementary Material S5, Section S2). Two of these can be monomers or homodimers (MARCHF1 and CLIC1). Besides these proteins, three other proteins can be homodimers, like butyrophilin subfamily 3 member A1, VDAC1 (homodimer and homotrimer) and RHAG (homodimer and heterotrimer). CD74 is a homotrimer, ryanodine receptor 3 can be a homotetramer or heterotetramer and MPEG1 is a homooligomer. TAP1 is a heterodimer, while the sodium channel protein type 4 subunit alpha can form a complex with a regulatory beta subunit. Cytochrome c oxidase subunit 1, STT3A and cytochrome b-245 heavy chain are parts of protein complexes.

The identified proteins were analyzed based on the Gene Ontology annotations. In what concerns the cellular components, the proteins with TM glycosylation sites were clustered based on cellular organelles. As can be seen in the Supplementary Material S5, Section S3, the largest number of proteins is found the endoplasmic reticulum, followed by the plasma membrane, mitochondrion, Golgi apparatus, nucleus, the lysosome and the vacuole. In what concerns the molecular functions and the biological processes involving these proteins, we observed a large diversity of functions and processes, which made the clustering more difficult. Therefore, we report the molecular functions and the biological processes clustered by parent GO terms involving at least three proteins (Tables S5.4 and S5.5 in Supplementary Material S5). The most prevalent molecular functions associated with the identified proteins are cation binding (seven proteins), transmembrane signaling receptor activity (five proteins) and protein binding (five proteins). The biological processes identified as involving a larger number of proteins are transmembrane transport (five proteins), transport (four proteins) and immune response (four proteins).

### 3.3. The Location of Identified Glycosylation Sites

In what concerns the glycosylation of proteins, it is known that O-GlcNAcylation mostly occurs in the cytoplasm, mitochondria and nucleus. The O-GlcNAc glycosylation sites could not be associated to a strict consensus sequence [10]. In the database search that we conducted, we identified 32 O-GlcNAc sites that were expected to be located in the membrane. In 10 cases, we could perform the location assessment of O-GlcNAc sites in 3D structures, with two of these sites having the result of being located outside of the membrane. Concerning the sites confirmed by 3D structures as located in the membrane, we showed that the side chains of Ser or Thr residues are located toward the interface with other TM helices, like in the case of Ser455 from cytochrome c oxidase subunit 1 (Figure 1b), or face the lipid membrane, like Thr371 in ammonium transporter Rh type A (Figure 2b). An interesting location of an O-GlcNAc site was seen in the case of Thr555 from phospholipid-transporting ATPase ABCA7 (Figure 2d) in which the side chain faces the lipid binding pocket. For 22 O-GlcNAc sites that we identified in the membrane, we could not do a location validation due to the lack of experimentally derived 3D structures. The search for O-GlcNac sites at the membrane interface led to the identification of two sites with cytoplasmatic localization: one is luminal, and one site is expected to be extracellular.

N-glycans can be attached to Asn-X-Ser/Thr sequences that can be accessed from the ER lumen. The efficiency of N-glycosylation is impacted by the “X” residue type and can be impaired if the folding of the protein hides the Asn-X-Ser/Thr motif. N-glycosylation was seen in the extracellular or luminal regions of proteins but not in their cytoplasmic regions [7]. Our study identified seven N-glycosylation sites that could be found in the membrane. One site was incorrectly annotated by GlyGen (Asn1162 in SCN4A), and two could not be verified in 3D structures (CLIC1 and GOLM2). In the remaining four cases, the analysis of 3D structures showed that these sites are actually extracellular. In the case of VDAC1, Asn238 is found in the TM region of a beta sheet, but it is located at the interface with the membrane, and its side chain faces the pore of the channel. The modeled glycan at that site also faces the pore of the channel. Several N-glycosylation sites predicted by Uniprot based on sequence analysis appeared to be located at the interface with the membrane. The location of most of these sites was confirmed at the interface with the membrane, but 3D structures showed no glycosylation at those sites. Only sodium channel protein type 10 subunit alpha is glycosylated at the predicted site, namely Asn1312, but the site is far from the membrane interface. Two of the predicted glycosylation sites were located on TM helices, which would make them inaccessible to glycosylating enzymes.

### 3.4. Samples Used for the Identification of Glycosylation Sites

In line with the pathophysiological relevance of glycosylation, the glycosylation sites located in TM regions that we identified (Table S1) resulted from studies performed mostly on cancer cell lines (especially HeLa cells—cervical cancer). There are also studies performed on non-tumor tissues, like human lung sections and isolated fibroblasts, human banked term placenta tissue or T cells. The TM glycosylation sites that we identified are mostly supported by a single study, but there are cases in which O-glycosylation sites are supported by up to nine studies involving non-tumor cells and tumor cell lines (for instance, the O-GlcNAcylation of Thr70 in VDAC3 is supported by nine studies). From the total O-glycosylation sites, 27 were identified in two studies performed on T cells [10,53]. Both studies reveal that O-glycosylation of proteins is involved in T cells activation [10,53]. It is interesting to note that TM O-glycosylation sites are found not only in activated T cells but also in unstimulated T cells or in both states (Table S1). This shows that TM O-glycosylation is not necessarily associated with a pathology or a particular condition like the activation of T cells.

The O-glycosylation sites that we located at the interface with the membrane were also identified mostly in studies performed on cancer cells (Table S2). At the same time, all sites were identified in activated T cells; two were identified in human lung sections and isolated fibroblasts, and one was identified in post mortem frozen brain tissue samples from Alzheimer patients.

### 3.5. Importance of Identified Glycosylation Sites

The results that we obtained on TM O-GlcNAc sites are interesting because these were determined experimentally by at least one study and were confirmed to be located in the membrane in most cases when 3D structures were available. Currently, there is a knowledge gap concerning the glycosylation of TM regions in proteins. Regarding the functional role of these TM O-glycosylation sites, by considering their location in between TM helices or towards the lipids in the membrane, we could suggest roles in folding, membrane insertion and interaction with the membrane. Still, the functional roles of these glycosylation sites in the TM and interface regions remain speculative and require experimental validation. The data that we obtained here are a valuable resource for future research, providing a starting point for studies on structural or functional implications of glycosylation in membrane regions.

## 4. Materials and Methods

The identification of proteins with O- or N-glycosylation sites in the TM regions or at the membrane interface was performed based on the datasets of proteins with glycosylation in the GlyGen database [51]. The lists were filtered for membrane proteins based on Uniprot [54] records of reviewed membrane proteins. The resulting lists are given in the Supplementary Material S6. Secondly, the residues expected to represent TM regions or membrane interface regions of previously identified proteins were retrieved from the Uniprot database [54], the subcellular location section, features—“transmembrane” annotations. The list of glycosylation sites from GlyGen was filtered based on the list of residues in topological domains from Uniprot, leading to the identification of O- and N-glycosylation sites in TM regions and at the membrane interface.

To validate the location of identified glycosylation sites, we retrieved the 3D structures of these proteins from Protein Data Bank [55]. The location of proteins’ residues relative to the membrane was retrieved from the OPM [56] and PDBTM [57] databases. In the case of proteins of unknown 3D structures, we identified the AlphaFold models [58] built with fair confidence (pLDDT > 70). For all proteins, based on the amino acids sequence, we additionally predicted the topological domains using the tools deepTMHMM—1.0 [59], TOPCONS [60], OCTOPUS [61], Philius [62], PolyPhobius [63], SCAMPI [64] and SPOCTOPUS [65]. Modeling of N-acetylglucosamine (NAG) glycans attached to the confirmed TM glycosylation sites was performed using CHARMM-GUI [66,67].

The database searches mentioned in the Results section and the information presented in Tables S1, S3, S4 and Supplementary Material S6 were reviewed in November 2024 and reflect the information status by this time period. Table S2 and Supplementary Material S5 were prepared in December 2024.

## 5. Conclusions

This study provides a detailed mapping of O- and N-glycosylation sites in protein TM regions and at membrane interfaces based on the information derived from database searches and structural validations. While glycosylation is traditionally associated with extracellular or intracellular regions, our results draw attention that glycosylation can occur in or near TM regions under specific conditions, supported by experimental evidence. Validation with available 3D structures confirmed the expected localization of most glycosylation sites. The exceptions that we identified highlighted discrepancies in database annotations. There is no information on the functional implications of glycans in these TM O-glycosylation sites, but we could speculate their influence in protein folding, membrane insertion, or interactions with lipid bilayers. This study supports the need for experimental validation to elucidate the biological significance of glycosylation in the membrane and offers a foundation for further exploration in structural and functional glycoproteomics.

## Figures and Tables

**Table 1:** Summary of reported and predicted glycosylation sites (GSs) identified in the TM region of proteins upon the database search *.

Protein	Uniprot ID	TM Region with GS	GS	Residue	Glycan Type	Paper PMID/ Reference	PDB Structure with TM Region
Reported O-glycosylation							
Butyrophilin subfamily 3 member A1	O00481	255–271	255	Thr	O-GlcNAcylation	29351928	-
Orexin/Hypocretin receptor type 1	O43613	120–140	129	Ser	O-GlcNAcylation	29351928	6TO7
			138	Ser	O-GlcNAcylation	29351928	
Cytochrome c oxidase subunit 1	P00395	447–478	455	Ser	O-GlcNAcylation	28510447, 29351928	5Z62
HLA class II histocompatibility antigen gamma chain	P04233	47–72	66	Thr	O-GlcNAcylation	29351928	-
Solute carrier family 25 member 16	P16260	191–211	195	Thr	O-GlcNAcylation	29351928	AF-P16260-F1-v4
			206	Ser	O-GlcNAcylation	29351928	
Non-selective voltage-gated ion channel VDAC1	P21796	202–211	211	Thr	O-GlcNAcylation	33214551, 30397120, 34725712, 23576270, 32119511, 28657654	5XDN
Dolichyl-diphosphooligosaccharide-protein glycosyltransferase subunit STT3A	P46977	120–138	124	Ser	O-GlcNAcylation	34846842, 34725712, 30059200, 29351928, 34019948, 35083852, 32119511, 35254053	6S7O
C-C chemokine receptor type 3	P51677	240–264	253	Thr	O-GlcNAcylation	29351928	7X9Y
			262	Ser	O-GlcNAcylation	29351928	
Ammonium transporter Rh type A	Q02094	363–383	371	Thr	O-GlcNAcylation	29351928	8CSX
Antigen peptide transporter 1	Q03518	54–76	63	Ser	O-GlcNAcylation	27655845, 30379171, 28510447	-
Putative sodium-coupled neutral amino acid transporter 11	Q08AI6	121–141	125	Ser	O-GlcNAcylation	29351928	AF-Q08AI6-F1-v4
			128	Ser	O-GlcNAcylation	29351928	
Protein patched homolog 1	Q13635	437–457	444	Ser	O-GlcNAcylation	30379171, 38253038	6DMB
Ryanodine receptor 3	Q15413	4187–4207	4201	Thr	O-GlcNAcylation	29351928, 30620550, 37217939, 38253038, 35254053	-
Solute carrier family 25 member 53	Q5H9E4	181–201	194	Ser	O-GlcNAcylation	30620550	AF-Q5H9E4-F1-v4
Protein O-mannosyl-transferase TMTC3	Q6ZXV5	318–338	337	Ser	O-GlcNAcylation	34725712, 38665916, 29351928	AF-Q6ZXV5-F1-v4
ATP-binding cassette sub-family A member 13	Q86UQ4	4536–4556	4539	Thr	O-GlcNAcylation	37217939, 29351928	-
			4544	Ser	O-GlcNAcylation	37217939, 29351928	
			4550	Thr	O-GlcNAcylation	37217939, 29351928	
Phospholipid-transporting ATPase ABCA7	Q8IZY2	550–570	555	Thr	O-GlcNAcylation	35008409, 29351928	8EDW
RING finger protein 175	Q8N4F7	104–121	107	Ser	O-GlcNAcylation	29351928	AF-Q8N4F7-F1-v4
Olfactory receptor 6K3	Q8NGY3	116–136	132	Thr	O-GlcNAcylation	29351928	AF-Q8NGY3-F1-v4
			133	Thr	O-GlcNAcylation	29351928	
E3 ubiquitin-protein ligase MARCHF1	Q8TCQ1	155–175	158	Ser	O-GlcNAcylation	29351928	-
			160	Thr	O-GlcNAcylation	29351928	
Polypeptide N-acetylgalactosaminyltransferase 14	Q96FL9	7–26	19	Thr	O-GlcNAcylation	29351928	-
Sideroflexin-1	Q9H9B4	229–249	232	Ser	O-GlcNAcylation	38665916, 34725712, 37217939, 35083852, 36240223, 33465208	AF-Q9H9B4-F1-v4
E3 ubiquitin-protein ligase MARCHF5	Q9NX47	238–258	238	Thr	O-GlcNAcylation	30379171	-
Voltage-dependent anion-selective channel protein 3	Q9Y277	69–76	70	Thr	O-GlcNAcylation	27655845, 23301498, 38665916, 34725712, 35132862, 35083852, 32870666, 34931806, 32119511	AF-Q9Y277-F1-v4
Reported N-glycosylation							
Chloride intracellular channel protein 1	O00299	26–46	42	Asn	N-linked	23090970, 24190977	1RK4
Cytochrome b-245 heavy chain	P04839	103–123	122	Asn	N-linked	24190977	8GZ3
Sodium channel protein type 4 subunit alpha	P35499	1160–1179	1162	Asn	N-linked	PDB annotations for 6AGF	6AGF
Non-selective voltage-gated ion channel VDAC1	P21796	231–238	238	Asn	N-linked	37074911	5XDN
Macrophage-expressed gene 1 protein	Q2M385	248–256	255	Asn	N-linked	37074911/24190977	6U2J
Protein GOLM2	Q6P4E1	15–35	31	Asn	N-linked	24190977	-
Protein patched homolog 1	Q13635	101–121	120	Asn	N-linked	PDB annotations for 6DMB, 6DMO, 6DMY –	6DMB
Predicted only O- and N-glycosylation							
Retinoic acid receptor responder protein 1	P49788	21–42	40	Ser	O-linked	27399812, 36213313, 37453717	-
Macrophage-expressed gene 1 protein	Q2M385	248–256	237	Thr	Not reported	GlyGen dataset GLY_001151	6U2J

* The columns represent: Protein—protein name according to Uniprot database; Uniprot ID—the entry ID in Uniprot database; TM region with GS—the TM regions comprising the glycosylation sites annotated according to Uniprot subcellular location; GS—glycosylation site reported in GlyGen database; Residue—the residues found at the glycosylation site reported in GlyGen database; Glycan type—the type of glycan annotated in GlyGen database at that site; Paper PMID/Reference—the PubMed IDs of papers reporting the glycosylation sites or other evidence supporting the glycosylation sites according to GlyGen; PDB structure with TM region—PDB structures comprising the TM regions with glycosylation sites discussed in the main text or AlphaFold models, in which the glycosylated TM regions were predicted with confidence.

**Table 2:** Proteins with reported O-glycosylation sites at residues found at the interface with the membrane (according to Uniprot annotations).

Protein	Uniprot ID	Glycosylation Site	Evidence PMIDs	Topological Domain Uniprot	Topological Domain Description	PDB
Lysosome-associated membrane glycoprotein 1	P11279	S381	23301498, 33214551, 8665916, 26374642, 34725712, 29351928, 34019948, 33465208, 35289036, 32119511, 36240223, 28657654	29–382	lumenal	AF-P11279-F1
Neuropeptides B/W receptor type 1	P48145	T70	29351928	62–72	cytoplasmic	AF-P48145-F1
Copper-transporting ATPase 2	P35670	T788	30379171, 29351928	786–919	cytoplasmic	7XUK, 7XUM, 7XUN, 7XUO, 8IOY
Plasma membrane calcium-transporting ATPase 4	P23634	T1051	28510447, 33465208, 38665916, 29351928	1016–1025	extracellular	AF-P23634-F1

**Table 3:** Proteins of known 3D structures with predicted N-glycosylation sites at residues found at the interface with the membrane (according to Uniprot annotations).

Protein	Uniprot ID	Glycosylation Site	Topological Domain Uniprot	Topological Domain Description	PDB	Glycan in PDB at the Site
Glycosylphosphatidylinositol anchor attachment 1 protein	O43292	N517	517–543	lumenal	7W72, 7WLD, 8IMX, 8IMY	no
Sodium channel protein type 10 subunit alpha	Q9Y5Y9	N1312	1312–1353	extracellular	7WFR, 7WEL, 7WE4, 7WFW	yes
Voltage-dependent R-type calcium channel subunit alpha-1E	Q15878	N1571	1561–1571	extracellular	7XLQ, 7YG5, 8EPL, 8EPM	no
Ammonium transporter Rh type A	Q02094	N355	354–362	extracellular	7UZQ, 7V0K, 7V0S, 8CRT, 8CS9, 8CSX, 8CTE	no
Malectin	Q14165	N268	29–269	lumenal	6S7T	no
Voltage-dependent N-type calcium channel subunit alpha-1B	Q00975	N1563	1556–1563	extracellular	7VFU, 7VFV, 7VFW, 7VFS, 7MIX, 7MIY	no
Sodium-dependent lysophosphatidylcholine symporter 1	Q8NA29	N240	222–241	extracellular	7OIX	no
Leukocyte surface antigen CD47	Q08722	N206	198–207	extracellular	7MYZ	no
Protein unc-93 homolog B1	Q9H1C4	N449	449–468	not annotated in Uniprot	7CYN, 7C76, 7C77	no
Toll-like receptor 4	O00206	N630	24–631	extracellular	5NAM, 5NAO	no

## Data Availability

All supporting data are given in Supplementary Materials.

## Supplementary Materials

The following supporting information can be downloaded at: https://www.mdpi.com/article/10.3390/ijms26010327/s1.
