# Supplementary material for: Mapping O- and N-Glycosylation in Transmembrane and Interface Regions of Proteins: Insights from a Database Search Study
Source: Int J Mol Sci. 2025 Jan 2;26(1):327. doi: 10.3390/ijms26010327 (PMC11720221; doi:10.3390/ijms26010327)
Supplement: Supplementary file 1 [file ijms-26-00327-s001.zip › Supplementary material S5.pdf]

Supplementary material S5

Statistical analysis on the dataset of proteins with TM glycosylation sites

S1. Frequency counts for the length of proteins: Figure S5.1 and Table S5.1

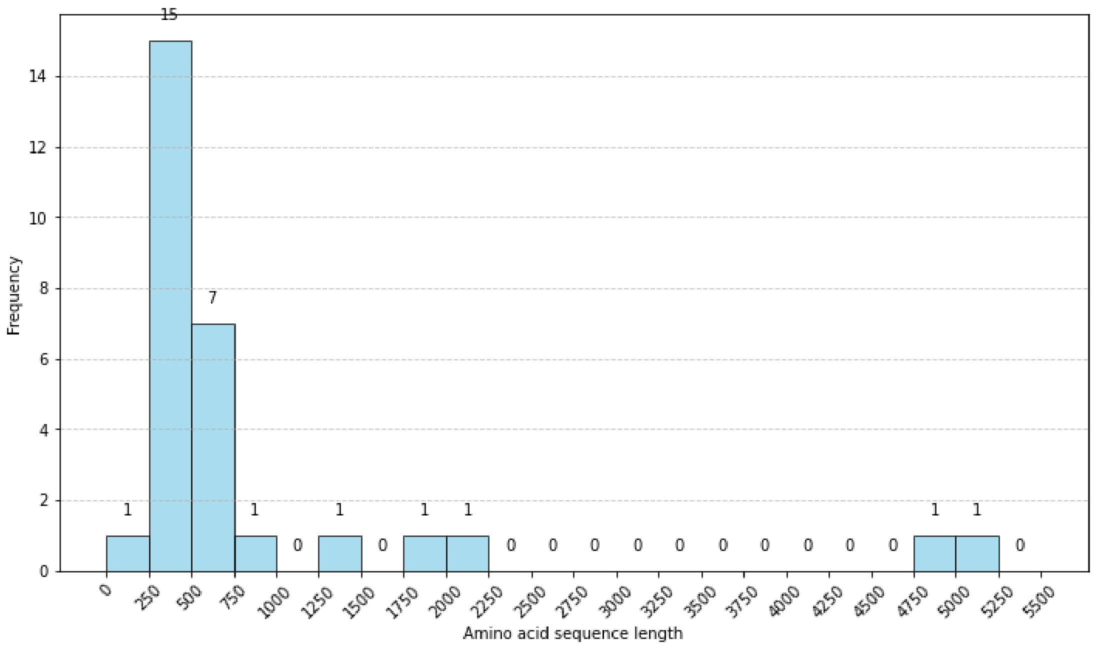

Figure S5.1. Histogram on the length of proteins with reported O- and N-glycosylation sites in TM regions (the list of proteins is in Table 1).

Table S5.1. Results of frquency counts conducted on the proteins with reported O- and N-glycosylation sites in TM regions (the list of proteins is in Table 1).

Sequence length (amino acids)	Count	Uniprot IDs
0-250	1	O00299
250-500	15	O43613; P04233; P16260; P21796; P51677; Q02094; Q08AI6; Q5H9E4; Q8N4F7; Q8NGY3; Q8TCQ1; Q9H9B4; Q9NX47; Q9Y277; Q6P4E1
500-750	7	O00481; P00395; P46977; Q03518; Q96FL9; P04839; Q2M385
750-1000	1	Q6ZXV5
1250-1500	1	Q13635
1750-2000	1	P35499
2000-2250	1	Q8IZY2
4750-5000	1	Q15413
5000-5250	1	Q86UQ4

S2. The oligomerization state of proteins: Figure S5.2 and Table S5.2.

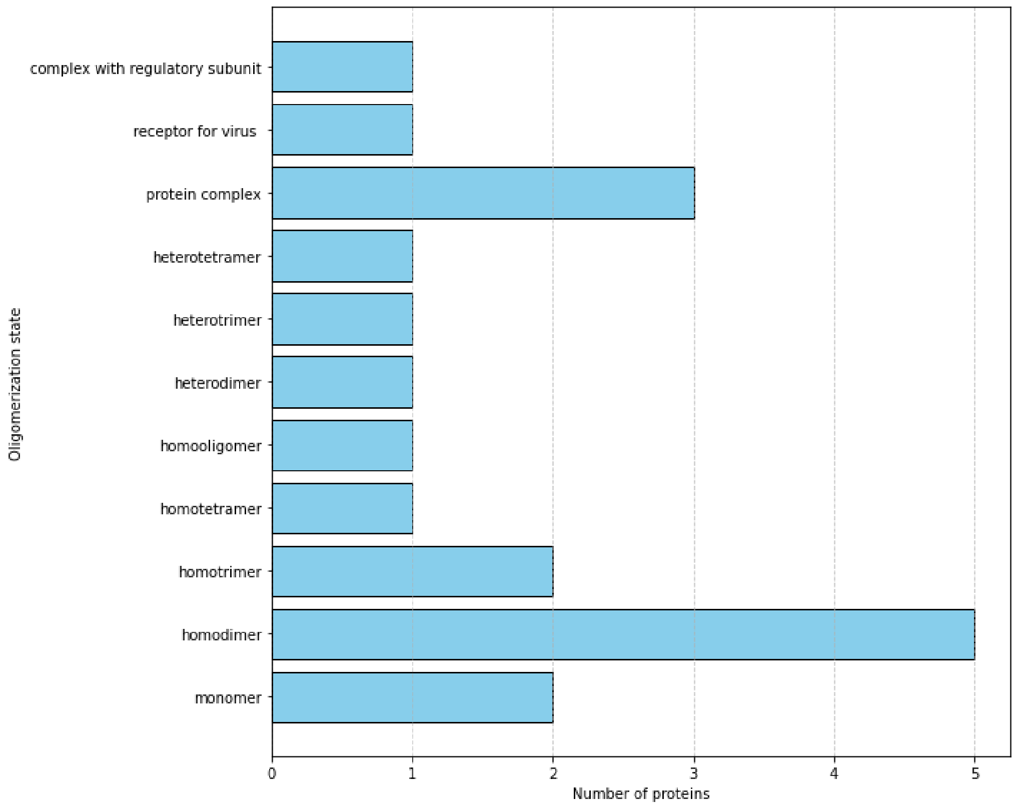

Figure S5.2. The number of proteins with reported TM glycosylation sites that can adopt different oligomeric states.

Table S5.2. The proteins with reported TM glycosylation sites that adopt different oligomeric states.

Oligomeric state	Count	Uniprot IDs
monomer	2	Q9NX47, O00299
homodimer	5	O00481, P21796, Q02094, Q9NX47, O00299
homotrimer	2	P04233, P21796
homotetramer	1	Q15413
homooligomer	1	Q2M385
heterodimer	1	Q03518
heterotrimer	1	Q02094
heterotetramer	1	Q15413
protein complex	3	P00395, P46977, P04839
receptor for virus	1	P51677
complex with regulatory subunit	1	P35499

S3. The cellular organelles with proteins presenting TM glycosylation sites: Figure S5.3 and Table S5.3

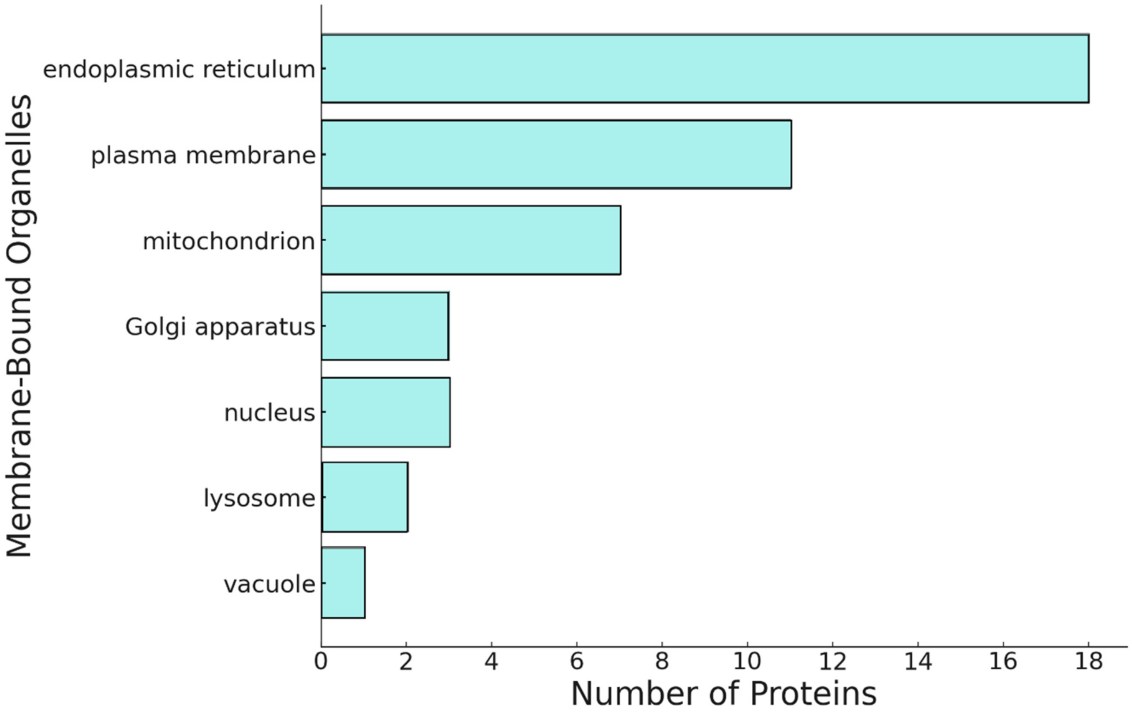

Figure S5.3. The distribution of proteins with TM glycosylation sites across membrane bound organelles.

Table S5.3. The count of proteins with TM glycosylation sites distributed in cellular organelles.

Organelle	Count	UniProt IDs
Endoplasmic Reticulum (Including Membrane)	18	P04233, P46977, Q03518, Q15413, Q6ZXV5, Q8IZY2, Q8N4F7, Q8TCQ1, Q9NX47, P04233, P46977, Q03518, Q8N4F7, Q8TCQ1, Q9NX47, Q03518, P04233, Q15413
plasma membrane [GO:0005886]	11	O00481, O43613, P04233, P21796, P51677, Q02094, Q13635, Q86UQ4, Q8IZY2, Q8NGY3, Q8TCQ1
mitochondrion [GO:0005739]	7	P00395, P16260, P21796, Q5H9E4, Q9H9B4, Q9NX47, Q9Y277
Golgi apparatus [GO:0005794]	3	Q8IZY2, Q8TCQ1, Q96FL9
nucleus [GO:0005634]	3	P04233, P21796, Q9Y277
lysosome [GO:0005764]	2	P04233, Q8TCQ1
vacuole [GO:0005773]	1	P04233

S4. The molecular functions of proteins with TM glycosylation sites: Table S5.4.

Table S5.4. The Molecular functions clustered by parent GO terms involving at least three of the proteins with reported glycosylation sites located in their TM regions.

Parent Term	GO Term Name	Protein Count	Uniprot IDs
GO:0043169	cation binding	7	P00395; P04839; P21796; P46977; Q03518; Q8N4F7; Q96FL9
GO:0004888	transmembrane signaling receptor activity	5	O43613; P04233; Q13635; Q8IZY2; Q8NGY3
GO:0005515	protein binding	5	O00481; P04233; P21796; Q13635; Q15413
GO:0005488	binding	4	O43613; Q13635; Q8NGY3; Q96FL9
GO:0032559	adenyl ribonucleotide binding	4	P21796; Q03518; Q86UQ4; Q8IZY2
GO:0035639	purine ribonucleoside triphosphate binding	4	P21796; Q03518; Q86UQ4; Q8IZY2
GO:0004842	ubiquitin-protein transferase activity	3	Q8N4F7; Q8TCQ1; Q9NX47
GO:0005102	signaling receptor binding	3	P04233; Q13635; Q8TCQ1
GO:0005253	monoatomic anion channel activity	3	O00299; P21796; Q9Y277
GO:0017111	ribonucleoside triphosphate phosphatase activity	3	Q03518; Q86UQ4; Q8IZY2
GO:0022857	transmembrane transporter activity	3	Q02094; Q03518; Q9H9B4
GO:0033218	amide binding	3	O43613; P04233; P21796
GO:0061659	ubiquitin-like protein ligase activity	3	Q8N4F7; Q8TCQ1; Q9NX47

S5. The biological processes involving proteins with TM glycosylation sites: Table S5.5.

Table S5.5. The biological processes clustered by parent GO terms involving at least three of the proteins with reported glycosylation sites located in their TM regions.

Parent Term	GO Name	Protein Count	Uniprot IDs
GO:0055085	transmembrane transport	5	P04839; P21796; Q02094; Q03518; Q08AI6
GO:0006810	transport	4	P16260; P21796; Q03518; Q86UQ4
GO:0006955	immune response	4	O00481; P04839; Q03518; Q2M385
GO:0002376	immune system process	3	P04233; P51677; Q8TCQ1
GO:0006950	response to stress	3	P00395; P04839; Q03518
GO:0007611	learning or memory	3	P21796; Q8IZY2; Q9Y277
GO:0009987	cellular process	3	O00299; P51677; Q03518
GO:0030001	metal ion transport	3	P35499; Q08AI6; Q15413
GO:0043410	positive regulation of MAPK cascade	3	O43613; P04233; Q8IZY2
GO:0070372	regulation of ERK1 and ERK2 cascade	3	O43613; P04233; Q8IZY2
GO:0098662	inorganic cation transmembrane transport	3	P35499; Q02094; Q15413
