# Supplementary material for: Mapping O- and N-Glycosylation in Transmembrane and Interface Regions of Proteins: Insights from a Database Search Study
Source: Int J Mol Sci. 2025 Jan 2;26(1):327. doi: 10.3390/ijms26010327 (PMC11720221; doi:10.3390/ijms26010327)
Supplement: Supplementary file 1 [file ijms-26-00327-s001.zip › Table_S1.pdf]

Table S1. List of reported and predicted glycosylation sites identified in the TM region of proteins with extended annotations.

The columns represent: Protein – protein name according to Uniprot database, Gene – the gene that encodes the protein, Uniprot ID – the protein ID from Uniprot database, TM region with glycosylation site (Evidence) – the TM regions comprising the glycosylation sites annotated according to Uniprot Subcellular location (criterion for manual assertion of TM region in Uniprot), Glycosylation site – glycosylation site reported in GlyGen database, Residue type – the residues found at the glycosylation site reported in GlyGen database, Glycan GlyTouCan Accesion – ID of glycan according to GlyTouCan repository, Glycan type – the type of glycan annotated in GlyGen database at that site, Paper PMID/Reference – the PubMed IDs of papers reporting the glycosylation sites or other evidence supporting the glycosylation sites according to GlyGen, Sample – sample used in the experimental study indicated by the PMID to investigate glycosylation; PDB structure with TM region – PDB structures comprising the TM regions with glycosylation sites discussed in the main text or Alphafold models in which the glycosylated TM regions were predicted with confidence.

Protein	Gene	Uniprot ID	TM region with glycosylation site (Evidence)	Fold	Glycosylation site	Residue type	Glycan GlyTouCan Accession	Glycan type	Reference PMID	Sample	PDB structure with TM region
**Reported O-glycosylation**											
Butyrophilin subfamily 3 member A1	BTN3A1	O00481	255-271 (sequence analysis)	helical	255	Thr	G49108TO	O-GlcNAcylation	29351928	Activated T cells	-
Orexin/Hypocretin receptor type 1	HCRTR1	O43613	120-140 (experiment)	helical	129	Ser	G49108TO	O-GlcNAcylation	29351928	Activated T cells	4ZJ8, 4ZJC, 6TO7, 6TOD, 6TOS, 6TOT, 6TP3, 6TP4, 6TP6, 6TQ4, 6TQ6, 6TQ7, 6TQ9, 6V9S
					138	Ser	G49108TO	O-GlcNAcylation	29351928	Activated T cells	
Cytochrome c oxidase subunit 1	MT-CO1	P00395		helical	455	Ser	G49108TO	O-GlcNAcylation	28510447	HeLa cells (cervical cancer)	

			447-478 (sequence similarity)						29351928	Activated T cells	5Z62
HLA class II histocompatibility antigen gamma chain	CD74	P04233	47-72 (sequence analysis)	helical	66	Thr	G49108TO	O-GlcNAcylation	29351928	Activated T cells	-
Solute carrier family 25 member 16	SLC25A16	P16260	191-211 (sequence analysis)	helical	195	Thr	G49108TO	O-GlcNAcylation	29351928	Activated T cells	AF-P16260-F1-v4
					206	Ser	G49108TO	O-GlcNAcylation	29351928	Activated T cells	
Non-selective voltage-gated ion channel VDAC1	VDAC1	P21796	202-211 (experiment)	Beta strand	211	Thr	G49108TO	O-GlcNAcylation	33214551	MCF-7 and MDA-MB-231 cells (breast cancer)	2JK4, 2K4T, 5JD4, 5XDN, 5XDO, 6G6U, 6G73, 6TIQ, 6TIR, 7QI2, 8J0O
									30397120	HepG2 cells (hepatocellular carcinoma)	
									34725712	U2OS cells (osteosarcoma)	
									23576270	Breast tumor specimens from patients	
									32119511	HEK293T (human embryonic kidney)	
									28657654	post-mortem frozen brain tissue samples from Alzheimer patients	
Dolichyl-diphosphooligosaccharide-	STT3A	P46977		helical	124	Ser	G49108TO	O-GlcNAcylation	34846842	HeLa cells (cervical cancer)	6S7O, 8B6L, 8PN9

protein glycosyltransferase subunit STT3A			120-138 (sequence similarity)						34725712	U2OS cells (osteosarcoma)	
									30059200	HeLa cells (cervical cancer)	
									29351928	Activated T cells	
									34019948	MCF-7, T47D and MDA-MB-231 cells (breast cancer)	
									35083852	HeLa cells (cervical cancer)	
									32119511	HEK293T (human embryonic kidney)	
									35254053	SW480 and SW620 cells (colorectal cancer)	
C-C chemokine receptor type 3	CCR3	P51677	240-264 (sequence analysis)	helical	253	Thr	G49108TO	O-GlcNAcylation	29351928	Unstimulated T cells	7X9Y
					262	Ser	G49108TO	O-GlcNAcylation	29351928	Unstimulated T cells	
Ammonium transporter Rh type A	RHAG	Q02094	363-383 (sequence analysis)	helical	371	Thr	G49108TO	O-GlcNAcylation	29351928	Activated T cells	7UZQ, 7V0K, 7V0S, 8CRT,

											8CS9, 8CSL, 8CSX, 8CTE
Antigen peptide transporter 1	TAP1	Q03518	54-76 (rules)	helical	63	Ser	G49108TO	O-GlcNAcylation	27655845	Activated T cells	-
									30379171	Hela cells (cervical cancer)	
									28510447	HeLa cells (cervical cancer)	
Putative sodium-coupled neutral amino acid transporter 11	SLC38A11	Q08AI6	121-141 (sequence analysis)	helical	125	Ser	G49108TO	O-GlcNAcylation	29351928	Unstimulated T cells	AF-Q08AI6-F1-v4
					128	Ser	G49108TO	O-GlcNAcylation	29351928	Unstimulated T cells	
Protein patched homolog 1	PTCH1	Q13635	437-457 (sequence analysis)	helical	444	Ser	G49108TO	O-GlcNAcylation	30379171	HeLa cells (cervical cancer)	6DMB, 6DMO, 6DMY, 6E1H, 6N7G, 6N7H, 6N7K, 6OEU, 6OEV, 6RMG, 6RVD
									38253038	human banked term placenta tissue	
Ryanodine receptor 3	RYR3	Q15413	4187-4207 (sequence analysis)	helical	4201	Thr	G49108TO	O-GlcNAcylation	29351928	Activated T cells	-
									30620550	293T cells (epithelial-like kidney cells)	
									37217939	BT-549 cells (breast cancer)	
									38253038	human banked term placenta tissue	

									35254053	SW480 and SW620 cells (colorectal cancer)	
Solute carrier family 25 member 53	SLC25A53	Q5H9E4	181-201 (sequence analysis)	helical	194	Ser	G49108TO	O-GlcNAcylation	30620550	293T cells (epithelial-like kidney cells)	AF-Q5H9E4-F1-v4
Protein O-mannosyl-transferase TMTC3	TMTC3	Q6ZXV5	318-338 (sequence analysis)	helical	337	Ser	G49108TO	O-GlcNAcylation	34725712	U2OS cells (osteosarcoma)	AF-Q6ZXV5-F1-v4
									38665916	human lung sections and isolated fibroblasts	
									29351928	Unstimulated T cells	
ATP-binding cassette sub-family A member 13	ABCA13	Q86UQ4	4536-4556 (sequence analysis)	helical	4539	Thr	G49108TO	O-GlcNAcylation	37217939	BT-549 cells (breast cancer)	-
									29351928	Unstimulated T cells	
					4544	Ser	G49108TO	O-GlcNAcylation	37217939	BT-549 cells (breast cancer)	
									29351928	Unstimulated T cells	
					4550	Thr	G49108TO	O-GlcNAcylation	37217939	BT-549 cells (breast cancer)	
									29351928	Unstimulated and activated T cells	
Phospholipid-transporting ATPase ABCA7	ABCA7	Q8IZY2	550-570 (sequence analysis)	helical	555	Thr	G49108TO	O-GlcNAcylation	35008409	CL1-1 and CL1-5 (lung adenocarcinoma)	8EDW, 8EE6, 8EEB, 8EOP
									29351928	Activated T cells	

RING finger protein 175	RNF175	Q8N4F7	104-121 (sequence analysis)	helical	107	Ser	G49108TO	O-GlcNAcylation	29351928	Activated T cells	AF-Q8N4F7-F1-v4
Olfactory receptor 6K3	OR6K3	Q8NGY3	116-136 (sequence analysis)	helical	132	Thr	G49108TO	O-GlcNAcylation	29351928	Activated T cells	AF-Q8NGY3-F1-v4
					133	Thr	G49108TO	O-GlcNAcylation	29351928	Activated T cells	
E3 ubiquitin-protein ligase MARCHF1	MARCHF1	Q8TCQ1	155-175 (sequence analysis)	helical	158	Ser	G49108TO	O-GlcNAcylation	29351928	Activated T cells	-
					160	Thr	G49108TO	O-GlcNAcylation	29351928	Activated T cells	
Polypeptide N-acetylgalactosaminyltransferase 14	GALNT14	Q96FL9	7-26 (sequence analysis)	helical	19	Thr	G49108TO	O-GlcNAcylation	29351928	Unstimulated and activated T cells	-
Sideroflexin-1	SFXN1	Q9H9B4	229-249 (sequence analysis)	helical	232	Ser	G49108TO	O-GlcNAcylation	38665916	human lung sections and isolated fibroblasts	AF-Q9H9B4-F1-v4
									34725712	U2OS cells (osteosarcoma)	
									37217939	BT-549 cells (breast cancer)	
									35083852	HeLa cells (cervical cancer)	
									36240223	PANC-1 cell (pancreatic ductal adenocarcinoma)	
									33465208	HeLa cells (cervical cancer)	
E3 ubiquitin-protein ligase MARCHF5	MARCHF5	Q9NX47	238-258 (sequence analysis)	helical	238	Thr	G49108TO	O-GlcNAcylation	30379171	HeLa cells (cervical cancer)	-
	VDAC3	Q9Y277			70	Thr	G49108TO		27655845	Activated T cells	

Voltage-dependent anion-selective channel protein 3			69-76 (sequence similarity)	Beta strand				O-GlcNAcylation	23301498	HEK293 cells (human embryonic kidney)	AF-Q9Y277-F1-v4
									38665916	human lung sections and isolated fibroblasts	
									34725712	U2OS cells (osteosarcoma)	
									35132862	Jurkat cells (immortalized T lymphocytes)	
									35083852	HeLa cells (cervical cancer)	
									32870666	HEK293 cells (human embryonic kidney)	
									34931806	Jurkat cells (immortalized T lymphocytes)	
									32119511	HEK293T (human embryonic kidney)	
**Reported N-glycosylation**											
Chloride intracellular channel protein 1	CLIC1	O00299	26-46 (sequence analysis)	Helical, after insertion into membrane	42	Asn	G80920RR	N-linked	23090970	Cell lines of different breast cancers in different stages	1RK4
									24190977	DLBCL cell lines (lymphoma)	

Cytochrome b-245 heavy chain	CYBB	P04839	103-123 (sequence analysis)	helical	122	Asn	Not reported	N-linked	24190977	DLBCL cell lines (lymphoma)	8GZ3, 8KEI, 8WEJ, 8X2L
Sodium channel protein type 4 subunit alpha	SCN4A	P35499	1160-1179 (experiment)	helical	1162	Asn	Not reported	N-linked	PDB annotations for 6AGF		6AGF
Non-selective voltage-gated ion channel VDAC1	VDAC1	P21796	231-238 (experiment)	Beta strand	238	Asn	G08609CW	N-linked	37074911	ccRCC cells (clear cell renal cell carcinoma)	2JK4, 2K4T, 5JD4, 5XDN, 5XDO, 6G6U, 6G73, 6TIQ, 6TIR, 7QI2, 8J0O
Macrophage-expressed gene 1 protein	MPEG1	Q2M385	248-256 (sequence similarity)	Beta strand	255	Asn	G62765YT/ not reported	N-linked	37074911<br>24190977	ccRCC cells (clear cell renal cell carcinoma)<br>DLBCL cell lines (lymphoma)	6U23, 6U2J, 6U2K, 6U2L, 6U2W
Protein GOLM2	GOLM2	Q6P4E1	15-35 (sequence analysis)	helical	31	Asn	Not reported	N-linked	24190977	DLBCL cell lines (lymphoma)	-
Protein patched homolog 1	PTCH1	Q13635	101-121 (sequence analysis)	helical	120	Asn	-	N-linked	PDB annotations for 6DMB, 6DMO, 6DMY		6DMB, 6DMO, 6DMY, 6E1H, 6N7G, 6N7H, 6N7K, 6OEU, 6OEV, 6RMG, 6RVD
**Predicted only O- and N-glycosylation**											

Retinoic acid receptor responder protein 1	RARRES1	P49788	21-42 (sequence analysis)	helical	40	Ser	O-linked	O-linked (Xyl...) (chondroitin sulfate) serine	27399812	samples published from both human urine and cerebrospinal fluid	-
									36213313	fibroblasts of apparently healthy individuals	
									37453717	human urine samples	
Macrophage-expressed gene 1 protein	MPEG1	Q2M385	248-256 (sequence similarity)	Beta stranded	237	Thr	O-linked	Not reported	Glygen dataset GLY_001151		6U23, 6U2J, 6U2K, 6U2L, 6U2W
